# Toll signaling promotes JNK-dependent apoptosis in *Drosophila*

**DOI:** 10.1186/s13008-020-00062-5

**Published:** 2020-03-10

**Authors:** Zhuojie Li, Chenxi Wu, Xiang Ding, Wenzhe Li, Lei Xue

**Affiliations:** 1 Institute of Intervention Vessel, Shanghai 10th People’s Hospital, Shanghai Key Laboratory of Signaling and Disease Research, School of Life Science and Technology, Tongji University, 1239 Siping Road, Shanghai, 200092 China; 2 College of Traditional Chinese Medicine, North China University of Science and Technology, 21 Bohai Road, Tangshan, 063210 China; 3 Zhuhai Interventional Medical Center, Zhuhai Precision Medical Center, Zhuhai People’s Hospital, Zhuhai Hospital Affiliated with Jinan University, Zhuhai, Guangdong 519000 China

**Keywords:** Cell death, *Drosophila*, Toll, JNK, ROS

## Abstract

**Background:**

Apoptosis plays pivotal roles in organ development and tissue homeostasis, with its major function to remove unhealthy cells that may compromise the fitness of the organism. Toll signaling, with the ancient evolutionary origin, regulates embryonic dorsal–ventral patterning, axon targeting and degeneration, and innate immunity. Using *Drosophila* as a genetic model, we characterized the role of Toll signaling in apoptotic cell death.

**Results:**

We found that gain of Toll signaling is able to trigger caspase-dependent cell death in development. In addition, JNK activity is required for Toll-induced cell death. Furthermore, ectopic Toll expression induces the activation of JNK pathway. Moreover, physiological activation of Toll signaling is sufficient to produce JNK-dependent cell death. Finally, Toll signaling activates JNK-mediated cell death through promoting ROS production.

**Conclusions:**

As Toll pathway has been evolutionarily conserved from *Drosophila* to human, this study may shed light on the mechanism of mammalian Toll-like receptors (TLRs) signaling in apoptotic cell death.

## Background

The type I trans-membrane receptor Toll was first identified in *Drosophila* for its role in establishing the dorsal–ventral axis at the early embryonic stage [1], and was subsequently determined as a key component of the innate immune response [2]. To date, nine Toll family members have been identified in fly and thirteen Toll-like receptors (TLRs) in mammals [3–6]. In *Drosophila*, Toll is activated by the cleaved cytokine Spätzle, and proceeds to the phosphorylation and degradation of the IκB factor Cactus through the MyD88-Tube-Pelle complex, eventually results in the release and translocation of two NF-kB factors Dorsal and Dorsal-related immunity factor (Dif) from cytoplasm to nucleus to activate the transcription of target genes [7]. Dorsal is required for the dorsal–ventral patterning during embryonic development, Dif is essential for the innate immunity in the adulthood, whereas both Dorsal and Dif are involved in the larval immune response [8–10].

The c-Jun N-terminal kinase (JNK) is a member of the highly conserved MAPKs family that plays pivotal roles in various cellular processes including apoptosis [11–15]. *basket* (*bsk*) encodes the sole *Drosophila* JNK that is phosphorylated and activated by the conserved upstream MAPK cascade, including the JNKK kinase dTAK1 and the JNK kinase Hemipterous (Hep) [16, 17]. *puckered* (*puc*), a target gene of JNK signaling, encodes a phosphatase that dephosphorylates and negatively regulates JNK activity [18]. In fly, JNK signaling plays an important role in programmed cell death [19], which is initiated by one or more of the pro-apoptotic genes *reaper* (*rpr*), *head involution defective* (*hid*) and *grim* [20], whose protein products bind to dIAP1 (*Drosophila* IAP-1) to release the initiator caspase Dronc (*Drosophila* NEDD2-like caspase) [21], which in turn activates the effector caspases Dcp-1 (Decapping protein 1) and Drice (Death related ICE-like caspase) [20]. JNK signaling can be activated by various extrinsic and intrinsic stress stimuli including oxidative stress generated by reactive oxygen species (ROS) [22–24], which is generated from partial reduction of oxygen, including hydroxyl radical, superoxide and hydrogen peroxide [25].

Besides the well-documented functions of Toll/NF-kB signaling in development and immunity, several reports suggest that Toll pathway is also required for cell death triggered by tumor necrosis factor (TNF) [26] or chromosomal instability (CIN) [27], yet the mechanism underlies Toll-induced cell death remain elusive. In this work, we employed *Drosophila* as an in vivo system and characterized that Toll signaling induces JNK-dependent apoptotic cell death via ROS production. Firstly, activation of Toll signaling induces apoptotic cell death in the developing wings and eyes. Secondly, depletion of JNK signaling suppresses Toll-induced apoptosis. Moreover, Toll signaling is able to trigger JNK pathway activation. Finally, Toll elicits JNK-dependent apoptosis via promoting ROS production.

## Results

### Toll signaling triggers cell death in *Drosophila* wing development

**Fig. 1:**
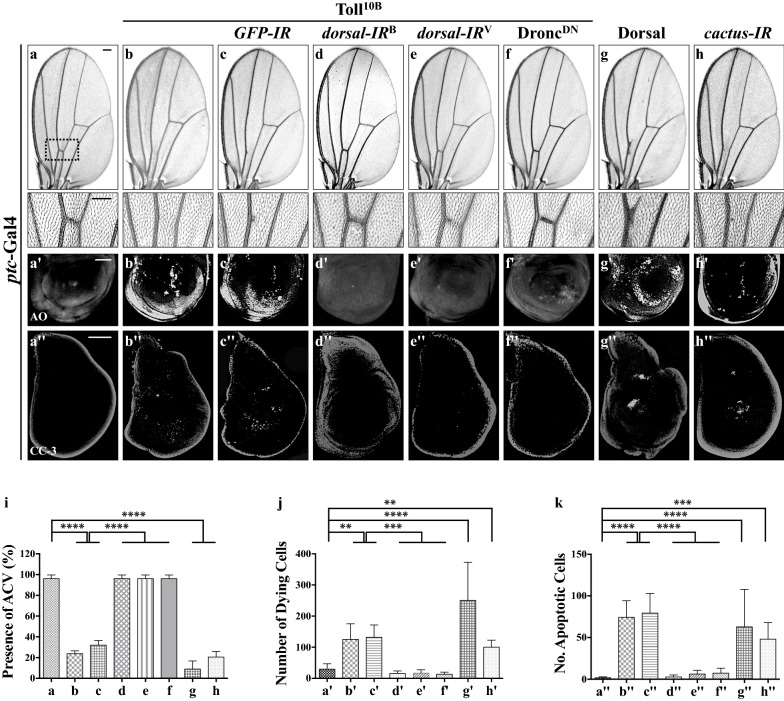
Activated Toll signaling triggers cell death in wing development. Light micrographs of *Drosophila* adult wings (**a**–**h**) and fluorescence micrographs of third instar larval wing discs (**a′**–**h′**, **a′′**–**h′′**) are shown. Compared with the *ptc*-Gal4 controls (**a**–**a′′**), *ptc *> Toll^10B^ induces a loss-of-ACV phenotype in adult wings (**b**), massive cell death (**b′**) and apoptosis (**b′′**) in third instar wing discs, all of which are suppressed by expressing two independent *dorsal* RNAi (**d**–**d′′**, **e**–**e′′**) or Dronc^DN^ (**f**–**f′′**), but not *GFP* RNAi (**c**–**c′′**). Expression of Dorsal or depletion of *cactus* also results in the loss-of-ACV phenotype in adult wings (**g**, **h**), and increased apoptotic cell death in third instar wing discs (**g′**, **g′′**, **h′**, **h′′**). The lower panels show high magnification view of the boxed areas in upper panels (**a**–**h**). For all wings, anterior is to the left and distal up. **i**–**k** Statistical analysis of the ACV phenotype in adult wings (**i**, n = 50 for each genotype), AO positive cell number (**j**, n = 9) and cleaved caspase-3 (CC-3) activity (**k**, n = 10) in wing discs are shown. Error bar indicates standard deviation. One-way ANOVA test was used to compute *P*-values, ****P < 0.0001, ***P < 0.001, **P < 0.01. *ptc*>Dorsal flies were reared at 20 °C to avoid lethality at 25 °C, while *ptc*>*cactus*-*IR* were reared at 29 °C to enhance the expression of RNAi. See Additional file 1 for detailed genotypes. Scale bar: 100 μm

**Fig. 2:**
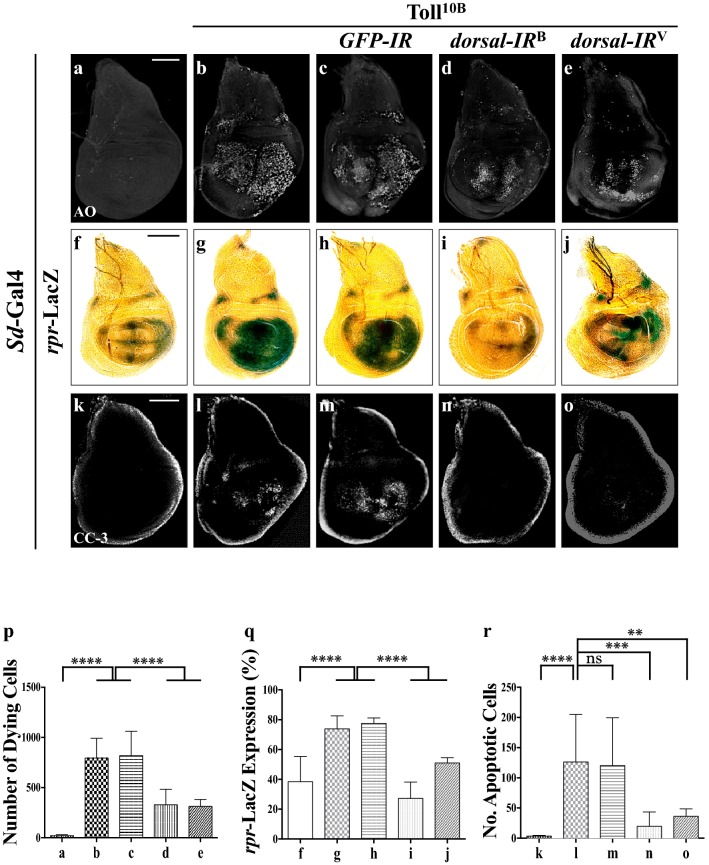
Toll signaling promotes apoptotic cell death. Fluorescence micrographs (**a**–**e**, **k**–**o**) and light micrographs (**f**–**j**) of third instar larval wing discs are shown. Compared with the controls (**a**, **f**, **k**), ectopic expression of Toll^10B^ in the wing pouch induces massive cell death (**b**), activates *rpr* transcription detected by X-Gal staining of a *rpr*-LacZ reporter (**g**), and promotes caspase activation indicated by cleaved caspase-3 (CC-3) antibody staining (**l**). These phenotypes are suppressed by depletion of *dorsal* (**d**, **e**, **i**, **j**, **n**, **o**), but not *GFP* (**c**, **h**, **m**). **p**–**r** Statistical analysis of cell death number (**p**, n = 8), X-Gal staining (**q**, n = 8) and CC-3 activity in wing discs (**r**, n = 9) are shown. Error bar indicates standard deviation. One-way ANOVA test was used to compute *P*-values, ****P < 0.0001, ***P < 0.001, **P < 0.01, ns is no significant difference. See Additional file 1 for detailed genotypes. Scale bar: 100 μm

Ectopic expression of Toll^10B^, an activated form of Toll, driven by *patched* (*ptc*)-Gal4 along the A/P compartment border (Additional file 1: Figure S1a) [28] (*ptc*>Toll^10B^) produces a loss of anterior cross vein (ACV) phenotype in the adult wings (Fig. 1a, b and quantified in Fig. 1i), which resembles the phenotype generated by expressing the cell death gene *grim* [26], implying a potential role of Toll signaling in promoting cell death in development. To validate this assumption, we performed Acridine Orange (AO) staining assay that detects dying cells [29], and observed massive cell death along the anterior/posterior (A/P) compartment boundary in 3rd instar larval wing discs (Fig. 1a′, b′ and quantified in Fig. 1j). Toll^10B^-induced loss-of-ACV phenotype and cell death were notably inhibited by expressing two independent *RNAi* lines of *dorsal* (Fig. 1d, e, d′, e′), which encodes the *Drosophila* NF-kB factor operating in the Toll pathway [30], but not *GFP* (Fig. 1c, c′). Furthermore, expression of Toll^10B^ in the wing pouch driven by *Scalloped* (*Sd*)-Gal4 (Additional file 1: Figure S1b) [31] results in enhanced cell death (Fig. 2a, b, p), which was suppressed by *RNAi*-mediated knockdown of *dorsal* (Fig. 2d, e), while *GFP RNAi* served as a negative control (Fig. 2c). A quantitative reverse transcription polymerase chain reaction (qRT-PCR) assay was performed to verify the knockdown efficiencies of the two *dorsal* RNAi lines (Additional file 1: Figure S2a). Consistently, over-expression of Dorsal produces a similar loss-of-ACV phenotype in the adult wing and cell death in the wing disc (Fig. 1g, g′), indicating that ectopic Toll-induced cell death depends on the canonical NF-kB pathway. Importantly, depletion of the IκB gene *cactus* also results in the loss of ACV and cell death (Fig. 1h, h′), suggesting a physiological function of the Toll/NF-kB pathway in developmental cell death.

### Toll signaling promotes caspase-mediated apoptotic cell death

Apoptosis in *Drosophila* is triggered by transcriptional up-regulation of one or more of three pro-apoptotic genes (*hid*, *rpr* and *grim*), and is mediated by the cleavage and activation of a group of cysteine proteases, termed caspases [32, 33]. *ptc*>Toll^10B^ triggers apoptosis visualized by anti-cleaved caspase-3 (CC-3) antibody staining (Fig. 1a′′, b′′ and quantified in Fig. 1k), which was suppressed by the expression of two independent *dorsal RNAi* (Fig. 1d′′, e′′), but remained unaffected by that of *GFP RNAi* (Fig. 1c′′). Moreover, expression of Dorsal or depletion of *cactus* also promotes apoptotic cell death (Fig. 1g′′, h′′). Intriguingly, Toll^10B^-induced apoptotic cell death and loss-of-ACV phenotype were efficiently blocked by expressing a dominant-negative form of the initiator caspase Dronc (Dronc^DN^) (Fig. 1f–f′′), suggesting Toll signaling induces caspase-dependent cell death. Consistently, expression of Toll^10B^ by *Scalloped* (*Sd*)-Gal4 up-regulates the transcription of the pro-apoptotic gene *rpr*, revealed by X-gal staining of a *rpr*-LacZ reporter, accompanied by enhances caspase activity (Fig. 2f, g, k, l, quantified in Fig. 2q, r) [34]. Both phenotypes were significantly impeded by the expression of *dorsal* RNAi (Fig. 2i, j, n, o), but not that of *GFP* (Fig. 2h, m). Thus, we conclude that Toll triggers NF-kB-mediated apoptotic cell death in *Drosophila.*

### Toll-induced cell death depends on JNK activity

**Fig. 3:**
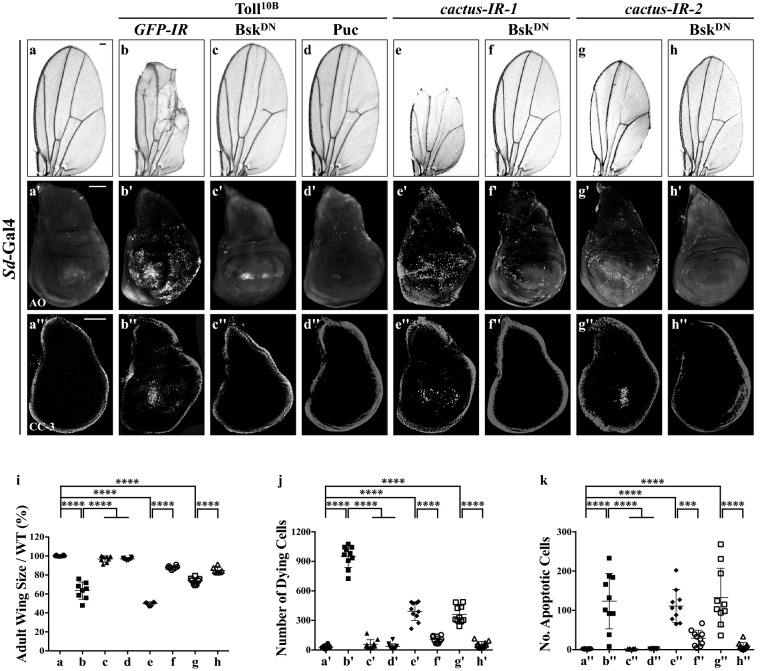
JNK signaling is required for Toll pathway-triggered cell death. Micrographs showing *Drosophila* adult wings (**a**–**h**) and third instar larval wing discs (**a′**–**h′**, **a′′**–**h′′**). Compared with the controls (**a**–**a′′**), ectopic expression of Toll^10B^ driven by *Sd*-Gal4 results in a small wing phenotype in adults (**b**), extensive cell death (**b′**) and apoptosis (**b′′**) in larval wing discs, which are suppressed by expressing a dominant-negative form of Bsk (**c**–**c′′**) or Puc (**d**–**d′′**). Depletion of *cactus* also produces a small wing phenotype in adults (**e**, **g**), massive cell death (**e′**, **g′**) and apoptosis (**e′′**, **g′′**) in larval wing discs, which are suppressed by expressing Bsk^DN^ (**f**–**f′′**, **h**–**h′′**). **i**–**k** Statistical analysis of the adult wing size/wild type (WT) (**i**, n = 8), cell death number (**j**, n = 10) and apoptosis (**k**, n = 10) in wing discs are shown. One-way ANOVA was used to compute *P*-values, ****P < 0.0001, ***P < 0.001. See Additional file 1 for detailed genotypes. Scale bar: 100 μm

Previous studies have suggested that the JNK signaling plays a critical role in the caspase-dependent cell death [19, 35–38]. To investigate whether JNK is required for Toll-induced cell death, we blocked JNK activity by expressing a dominant negative form of *Drosophila* JNK Bsk (Bsk^DN^) or Puc, an inhibitor of JNK kinase activity [18]. We found that *Sd*>Toll^10B^-induced adult wing blade reduction and apoptotic cell death in 3rd instar larval wing discs were soundly suppressed by expressing Bsk^DN^ or Puc (Fig. 3a–d, a′–d′, a′′–d′′ and quantified in Fig. 3i–k). Notably, depletion of *cactus* by two independent RNAi (Additional file 1: Figure S2b) was also sufficient to generate Bsk-dependent small wing phenotype and apoptotic cell death (Fig. 3e–h, e′–h′, e′′–h′′). Furthermore, *ptc*>Toll^10B^- or *ptc*>*cactus*-*IR*-triggered loss-of-ACV phenotype was fully suppressed by blocking JNK activity (Additional file 1: Figure S3). Taken together, these data proved that JNK activity is indispensable for ectopic or physiological Toll/NF-kB pathway-induced cell death.

### Elevated Toll signaling activates JNK pathway

**Fig. 4:**
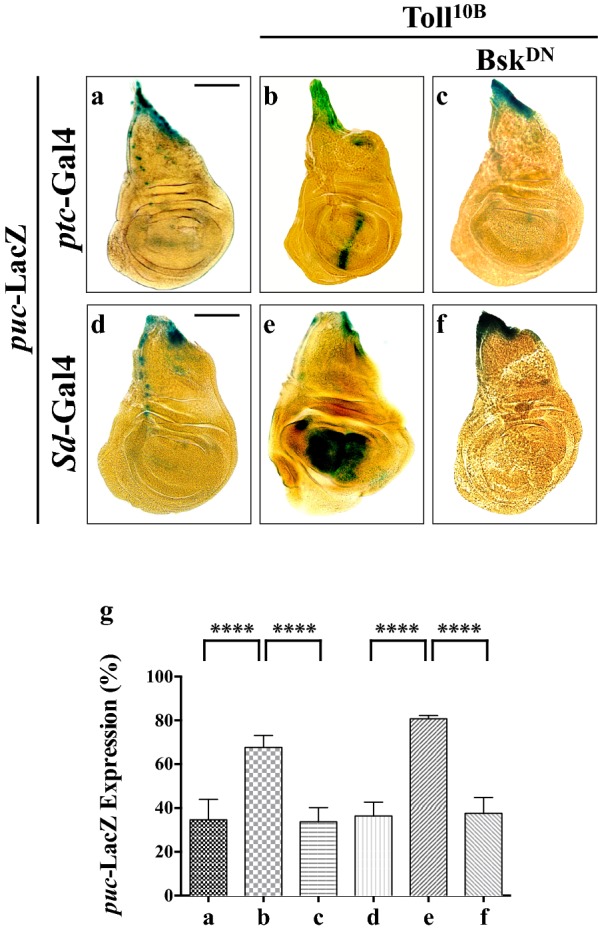
Toll activates JNK signaling. Light micrographs of third instar larval wing discs with X-Gal staining (**a**–**f**) are shown. Compared with the *ptc*-Gal4 and *Sd*-Gal4 controls (**a**, **d**), ectopic expression of Toll^10B^ up-regulates *puc*-LacZ expression (**b**, **e**), which is blocked by expressing Bsk^DN^ (**c**, **f**). **g** Statistical analysis of X-Gal staining (n = 8) is shown. One-way ANOVA was used to compute *P*-values, ****P < 0.0001. See Additional file 1 for detailed genotypes. Scale bar: 100 μm

Given that JNK activity is required for Toll-induced cell death, we hypothesized that Toll may induce the activation of JNK pathway. To monitor JNK pathway activation, we examined the expression of *puc*-LacZ, a widely accepted reporter for JNK signaling [18]. In support of the assumption, we found that *puc*-LacZ expression was significantly activated by ectopic Toll^10B^ expression driven by *ptc*-Gal4 or *Sd*-Gal4 in a Bsk-dependent manner (Fig. 4a–f and quantified in Fig. 4g). Moreover, depletion of *cactus* also up-regulated *puc*-LacZ expression (Additional file 1: Figure S4), suggesting both ectopically and physiologically elevated Toll signaling are sufficient to trigger JNK pathway activation, which in turn activates apoptotic cell death.

### JNK is required for Toll-induced cell death in *Drosophila* eye development

**Fig. 5:**
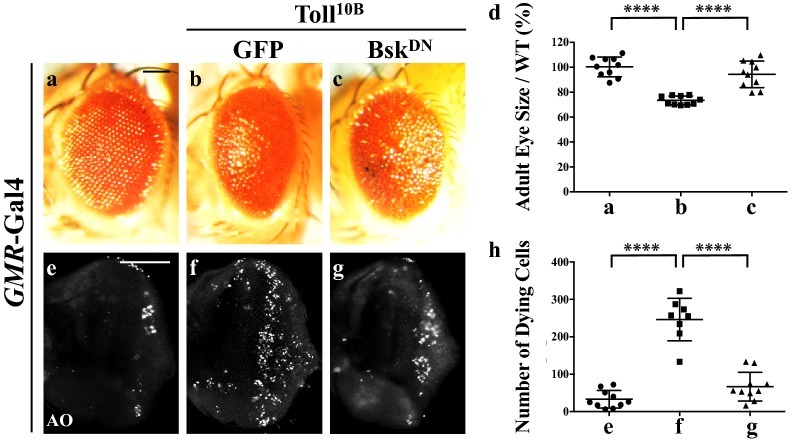
Toll triggers JNK-dependent cell death in eye development. Light micrographs of *Drosophila* adult eyes (**a**–**c**) and fluorescent micrographs of third instar larval eye discs (**e**–**g**) are shown. Compared with the *GMR*-Gal4 controls (**a**, **e**), Toll^10B^-induced small eye phenotype (**b**) and cell death (**f**) are suppressed by expressing Bsk^DN^ (**c**, **g**). **d**, **h** Statistical analysis of the adult eye size/wild type (WT) (n = 10) and cell death number in eye discs (**e**, n = 10; **f**, n = 8; **g**, n = 10) are shown. One-way ANOVA was used to compute *P*-values, ****P < 0.0001. See Additional file 1 for detailed genotypes. Scale bar: 100 μm

To investigate whether Toll signaling triggers JNK-dependent cell death in other cellular contexts, we expressed Toll^10B^ by *Glass Multimer Reporter* (*GMR*)-Gal4, which expresses in all cell types posterior to the morphogenetic furrow (MF) in the developing eye (Additional file 1: Figure S1c) [39]. We observed extensive cell death posterior to the MF in third instar larval eye discs (Fig. 5e, f and quantified in Fig. 5h), and size reduction of adult eyes (Fig. 5a, b and quantified in Fig. 5d). Both phenotypes were suppressed by expressing Bsk^DN^ (Fig. 5c, g), suggesting that Toll induces JNK-mediated cell death in a non-tissue-specific manner.

### Toll activates JNK-mediated cell death through ROS

**Fig. 6:**
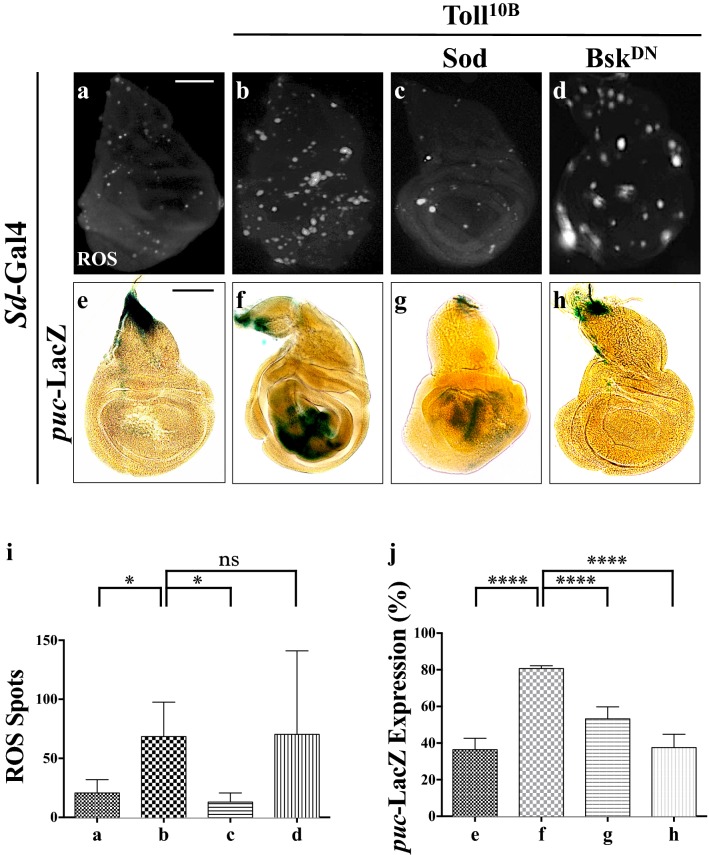
Toll induces ROS-dependent JNK activation and apoptosis. Third instar larval wing discs showing ROS level (**a**–**d**) and *puc*-LacZ expression (**e**–**h**). Compared with the control (**a**), ectopic expression of Toll^10B^ promotes ROS production (**b**), which is suppressed by expressing Sod (**c**), but not Bsk^DN^ (**d**). Compared with the control (**e**), Toll^10B^-induced JNK pathway activation (**f**) is suppressed by expressing Sod (**g**) or Bsk^DN^ (**h**). **i**, **j** Statistical analysis of the ROS spots (**a**, n = 10; **b**, n = 7; **c**, n = 10; **d**, n = 9) and X-Gal expression (n = 8) are shown. Kruskal–Wallis test and one-way ANOVA were used to compute *P*-values, ****P < 0.0001, *P < 0.05 and ns indicates not significant. See Additional file 1 for detailed genotypes. Scale bar: 100 μm

JNK signaling could be activated by reactive oxygen species (ROS)-mediated oxidative stress in *Drosophila* [23]. To address whether ROS is involved in Toll-induced JNK-dependent cell death, we examined ROS level in 3rd instar wing discs. Compared with the *Sd*-Gal4 control (Fig. 6a), Toll^10B^ expression strongly raised ROS production (Fig. 6b and quantified in Fig. 6i), which was suppressed by expression of Sod (Fig. 6c), a superoxide dismutase enzymes that eliminates oxygen radicals [23], but not by Bsk^DN^ (Fig. 6d), suggesting Toll-induced ROS production is independent of JNK. To verify the results, we assessed the expression of *gstD1* (*Glutathione S transferase D1*), which encodes a detoxification enzyme that responds to oxidative stress [40, 41]. We found that ectopic Toll^10B^ was sufficient to increase the gstD1 mRNA level as measured by qRT-PCR, which was suppressed by expressing Sod, but not Bsk^DN^ (Additional file 1: Figure S5a). Intriguingly, Toll^10B^-induced *puc*-LacZ expression were significantly impeded by expressing Sod (Fig. 6e–g and quantified in Fig. 6j), while Bsk^DN^ served as a positive control (Fig. 6h). Collectively, our data suggest that Toll signaling promotes ROS production, which activates JNK-mediated apoptotic cell death.

## Discussion

*Drosophila* has been widely accepted as an excellent model organism to dissect the roles of various signaling pathways in regulating the apoptotic program for the last two decades [42, 43]. While the functions of Toll pathway in embryonic dorsal–ventral patterning and innate immunity have been extensively studied, much less is known about its cell death functions in development, and the underlying mechanisms remain largely elusive. Necroptosis is a type of programmed cell death, characterized by membrane swell and rupture, which is mediated by receptor-interacting protein kinase 1 (RIPK1) and RIPK3, but independent of caspases activity [44]. In vitro studies show activation of mammalian Toll-like receptors (TLRs) can trigger necroptotic cell death through RIPK1, RIPK3, and pseudokinase mixed lineage kinase-domain-like (MLKL) complex [45, 46]. Intriguingly, inhibition of JNK with SP600125 restricts TLRs-induced necroptosis in macrophages, whereas loss of JNK by short-interfering RNA (siRNA) augments TLRs-induced necroptotic cell death, suggesting a dual role for JNK in regulating necroptosis [47]. Recent studies in *Drosophila* suggest that Toll signaling, consisting of the Toll ligand Spätzle, several Toll-related receptors and NF-kB factors, is required for apoptotic cell death of loser cell elimination during epithelial cell competition, which up-regulates the expression of pro-apoptotic genes, yet this function of Toll signaling is independent of the JNK activity [48–51]. In addition, Toll signaling also plays pivotal roles in cell number plasticity during the nervous system development [52]. *Drosophila* neurotrophin (NT) ligands (including Spätzle, Spätzle2 and Spätzle5), combined with distinct Toll receptors, can switch from promoting cell survival to death in the central nervous system (CNS) via a three-tier mechanism [53]. For example, Toll-6 promotes neuronal survival via MyD88/NF-κB in the embryonic CNS but neuronal death via Wek/Sarm/JNK in the pupal CNS. However, whether NTs function in cooperation with TLRs to mediate neuronal survival/death in mammals is still unclear. Previous study also suggested that Toll-JNK is required for chromosomal instability (CIN)-triggered cell death in proliferating *Drosophila* larval tissue [27], yet whether activated Toll signaling is sufficient to promote JNK-mediated cell death, and the underlying mechanism from Toll to JNK remains unknown.

Toll signaling can be activated in response to Gram positive bacteria, fungi and viruses in both Drosophila and mammals [2, 54–58]. In the present study, we first found that ectopic expression of Toll triggers JNK-mediated apoptotic cell death, yet in this scenario, Toll is expressed at a much higher level than that induced by injection of *Staphylococcus aureus* (*S. aureus*), a Gram-positive bacterium (Additional file 1: Figure S6a). To overcome this artificial activation which may not implicate a physiological relevance, we depleted *cactus* encoding the *Drosophila* IκB factor, and confirmed that physiological activation of Toll signaling is sufficient to induce JNK-dependent apoptosis in development. Furthermore, we show that Toll signaling triggers JNK activation via promoting the production of reactive oxygen species (ROS), yet the mechanism by which Toll signaling activates ROS production remains unclear, which merits further investigation. Consistent with our findings, mammalian TLRs was reported to induce the production of pro-inflammatory mediators upon pathogen invasion, which act as secondary messengers to regulate oxidative stress [59]. Up-regulated expression of inflammatory regulators, such as inducible nitric oxide synthase (iNOS), resulted in high levels of ROS [60]. Given that ROS production is closely associated with mitochondria dysfunction, a recent study found that TLRs recruited mitochondria to macrophage phagosomes and augmented ROS production [61].

## Conclusions

In this study, we report that Toll signaling induces ROS-mediated JNK-dependent apoptotic cell death in vivo. First, we characterized a physiological function of the Toll/NF-kB signaling in developmental cell death. In addition, Toll-induced cell death depends on JNK activity. Furthermore, Toll signaling is sufficient to trigger JNK pathway activation. Finally, we provide evidence that Toll activates JNK-mediated apoptosis through ROS production. Thus, our study provided the first in vivo evidence that Toll signaling promotes JNK-dependent apoptosis via ROS production.

## Materials and methods

### *Drosophila* strains

Flies were raised on a standard cornmeal and ager medium at 25 °C unless otherwise indicated. Fly strains used in this work include: *ptc*-Gal4, *Sd*-Gal4, *GMR*-Gal4, *UAS*-GFP [62], *act*-Gal4, *UAS*-*GFP*-*IR* [63], *UAS*-Toll^10B^ [26], *UAS*-Dronc^DN^, *UAS*-Bsk^DN^, *UAS*-Puc, *puc*^E69^ and *rpr*-LacZ [64] were previously described. *UAS*-*dorsal*-*IR* (27650) and *UAS*-Sod1 (24750) were obtained from the Bloomington stock center, *UAS*-*dorsal*-*IR* (45998) was obtained from the VDRC center, *UAS*-*cactus*-*IR*-*1* (5848R-3) and *UAS*-*cactus*-*IR*-*2* (5848R-1) were obtained from the NIG-FLY center, and *dl*^*d05894*^ was obtained from the Exelixis collection at Harvard.

### Microbial injection

*Staphylococcus aureus* (*S. aureus*) strain was obtained from Prof. Wei Zuo at Tongji University. For experiment, the bacterial culture diluted with PBS to 1 × 10^13^ cells/ml. Third instar larvae were washed with PBS. l μl bacterial suspension of *S. aureus* was injected into the larva body with a sharp needle. Treated larvae were placed on the medium at 25 °C. mRNA level was monitored by qRT-PCR 4 h post infection.

### qRT-PCR

Eastep Super (Shanghai Promega) was used to isolate total RNA from ten third instar larvae of indicated genotypes, and qRT-PCR was performed using SYBR Green PCR Premix Kit (TaKaRa). Primers used were as follows:

For *rp49* FP: TCTCCTTGCGCTTCTTGGA

RP: TACAGGCCCAAGATCGTGAA

For *dl* FP: ATCCGTGTGGATCCGTTTAA

RP: AATCGCACCGAATTCAGATC

For *cactus* FP: CTCACTAGCCACTAGCGGTAA

RP: CCCGAATCACTGGTTTCGTTT

For *Toll* FP: AATCCCACGTTTAGGCTAACCA

RP: CCTCACCGATCCGCAACTT

For *gstD1* FP: CGCGCCATCCAGGTGTATTT

RP: CTGGTACAGCGTTCCCATGT

### AO staining

Eye and wing discs were dissected from third-instar larvae in 0.1% PBST (phosphate-buffered saline (PBS) + 0.1% Tween-20) and incubated in 1 × 10^−5^ M AO for 5 min at room temperature prior to imaging [65].

### Immunostaining

Antibody staining was performed by standard procedures for imaginal discs [66]. Rabbit anti-Cleaved Caspase-3 (1:400, Cell Signaling Technology, CST, Cat # 9661, Danvers, MA, USA) was used as a primary antibody, and goat anti-rabbit CY3 (1:1000, Life technologies, Cat # A10520) was used as a secondary antibody.

### X-gal staining

Wing discs were dissected from third instar larvae in PBST (PBS + 0.1% Tween-20) and stained for β-galactosidase as described [67].

### ROS detection

The level of ROS was measured by CellROX (Life Technologies, C10443). Wing discs were dissected from third instar larvae, incubated in 5 μM CellROX for 30 min at 37 °C, rinsed in PBS, fixed in 3.7% formaldehyde for 5 min, and mounted in PBS for imaging [68].

### Image and quantification of fly eyes and wings

Images of fly eyes and wings were prepared as described [69]. Briefly, 3-day-old flies were collected and frozen at − 80 °C for more than 12 h. When taking pictures, flies were unfrozen at room temperature and placed on 1% agarose plate. Light images of eye were taken by OLYMPUS stereo microscope SZX16 (Olympus Corporation, Shinjuku, Tokyo, Japan). Wings were dissected and placed on slide with alcohol/glycerol (1:1) medium. Light images of wing were taken by OLYMPUS BX51 microscope. Adobe Photoshop 2014 was used to measure the size of fly wings and eyes on the images.

## Supplementary information

**Additional file 1: Figure S1.** The expression patterns of *ptc*-Gal4, *Sd*-Gal4 and *GMR*-Gal4. Fluorescence micrographs of third instar larval wing (**a**, **b**) and eye discs (**c**) are shown. Expression region of *ptc*-Gal4 (**a**), *Sd*-Gal4 (**b**) and *GMR*-Gal4 (**c**) are labeled by the *UAS*-GFP reporter. Scale bar: 100 μm. **Figure S2.** The knock-down efficacies of *dorsal* and *cactus* RNAi lines. (**a** and **b**) Expression of two independent *dorsal* RNAi and *cactus* RNAi significantly decrease the level of *dorsal* mRNA and *cactus* mRNA, as measured by qRT-PCR. Total RNA of *Drosophila* third instar larval wing discs (n = 10, in each group) was extracted and normalized for cDNA synthesis. Error bar indicates standard deviation. One-way ANOVA test was used to compute *P*-values, ****P < 0.0001, ***P < 0.001, **P < 0.01, *P < 0.05. **Figure S3.** JNK is required for Toll/NF-kB signaling impaired ACV development. Light micrographs of *Drosophila* adult wings (**a**-**f**) are shown. Compared with the *ptc*-Gal4 control (**a**), Toll^10B^-induced loss-of-ACV phenotype in adult wings (**b**), is blocked by expressing Bsk^DN^ (**c**) or Puc (**d**). Depletion of *cactus* also produces a loss-of-ACV phenotype (**e**), which is suppressed by expressing Bsk^DN^ (**f**). The lower panels show high magnification view of the boxed areas in upper panels (**a**-**f**). (**g**) Statistical analysis of ACV phenotype in adult wings (n = 45 for each genotype) is shown. Error bar indicates standard deviation. One-way ANOVA test was used to compute *P*-values, ****P < 0.0001. Scale bar: 100 μm. **Figure S4.** JNK pathway is up-regulated by physiological activation of Toll signaling. Light micrographs of third instar wing discs with X-Gal staining (**a**-**c**) are shown. Compared with the *Sd*-Gal4 control (**a**), elevating endogenous Toll signaling by knockdown of *cactus* (**b** and **c**) up-regulates *puc*-LacZ expression. (**d**) Statistical analysis of X-Gal staining (n = 8) is shown. One-way ANOVA was used to compute *P*-values, ****P < 0.0001. Scale bar: 100 μm. **Figure S5.** Toll regulates the stress response gene *gstD1*. (**a**) Histogram showing the level of *gstD1* mRNA as measured by qRT-PCR. Error bar represents standard deviation from three independent experiments. One-way ANOVA was used to compute *P*-values, ***P < 0.001, ns indicates not significant. **Figure S6.** Evaluate the level of *Toll* expression. (**a**) Histogram showing the level of *Toll* mRNA as measured by qRT-PCR. Error bar represents standard deviation from three independent experiments. One-way ANOVA was used to compute *P*-values, ****P < 0.0001, **P < 0.01.

## Data Availability

The data that support the findings of this study are available from the corresponding author upon reasonable request.

## Abbreviations

JNK: c-Jun N-terminal kinase
MAPK: Mitogen-activated protein kinase
ROS: Reactive oxygen species
NF-kB: Nuclear factor kB
TLR: Toll-like receptor
ACV: Anterior cross vein
CIN: Chromosomal instability
qRT-PCR: Quantitative reverse transcription polymerase chain reaction
