# Supplementary material for: Toll signaling promotes JNK-dependent apoptosis in Drosophila
Source: Cell Div. 2020 Mar 10;15:7. doi: 10.1186/s13008-020-00062-5 (PMC7063707; doi:10.1186/s13008-020-00062-5)
Supplement: Supplementary file 1 — Additional file 1: Figure S1. The expression patterns of ptc-Gal4, Sd-Gal4 and GMR-Gal4. Fluorescence micrographs of third instar larval wing (a, b) and eye discs (c) are shown. Expression region of ptc-Gal4 (a), Sd-Gal4 (b) and GMR-Gal4 (c) are labeled by the UAS-GFP reporter. Scale bar: 100 μm. Figure S2. The knock-down efficacies of dorsal and cactus RNAi lines. (a and b) Expression of two independent dorsal RNAi and cactus RNAi significantly decrease the level of dorsal mRNA and cactus mRNA, as measured by qRT-PCR. Total RNA of Drosophila third instar larval wing discs (n = 10, in each group) was extracted and normalized for cDNA synthesis. Error bar indicates standard deviation. One-way ANOVA test was used to compute P-values, ****P < 0.0001, ***P < 0.001, **P < 0.01, *P < 0.05. Figure S3. JNK is required for Toll/NF-kB signaling impaired ACV development. Light micrographs of Drosophila adult wings (a-f) are shown. Compared with the ptc-Gal4 control (a), Toll10B-induced loss-of-ACV phenotype in adult wings (b), is blocked by expressing BskDN (c) or Puc (d). Depletion of cactus also produces a loss-of-ACV phenotype (e), which is suppressed by expressing BskDN (f). The lower panels show high magnification view of the boxed areas in upper panels (a-f). (g) Statistical analysis of ACV phenotype in adult wings (n = 45 for each genotype) is shown. Error bar indicates standard deviation. One-way ANOVA test was used to compute P-values, ****P < 0.0001. Scale bar: 100 μm. Figure S4. JNK pathway is up-regulated by physiological activation of Toll signaling. Light micrographs of third instar wing discs with X-Gal staining (a-c) are shown. Compared with the Sd-Gal4 control (a), elevating endogenous Toll signaling by knockdown of cactus (b and c) up-regulates puc-LacZ expression. (d) Statistical analysis of X-Gal staining (n = 8) is shown. One-way ANOVA was used to compute P-values, ****P < 0.0001. Scale bar: 100 μm. Figure S5. Toll regulates the stress response gene gstD1. [file 13008_2020_62_MOESM1_ESM.docx]

**Toll signaling promotes JNK-dependent apoptotic cell death in *Drosophila***

Zhuojie Li^1,#^, Chenxi Wu^1,2,#^, Xiang Ding^1^, Wenzhe Li^1,*^ and Lei Xue^1,3*^

^1^ Institute of Intervention Vessel, Shanghai 10th People's Hospital, Shanghai Key Laboratory of Signaling and Disease Research, School of Life Science and Technology, Tongji University, 1239 Siping Road, Shanghai 200092, China

^2^ College of Traditional Chinese Medicine, North China University of Science and Technology, 21 Bohai Road, Tangshan 063210, China

^3^ Zhuhai Interventional Medical Center, Zhuhai Precision Medical Center, Zhuhai People's Hospital, Zhuhai Hospital Affiliated with Jinan University, Zhuhai, Guangdong 519000, China

^#^ These authors contribute equally to this work

^*^ Correspondence: [lei.xue@tongji.edu.cn](mailto:lei.xue@tongji.edu.cn), [lwz@tongji.edu.cn](mailto:lei.xue@tongji.edu.cn)

**Additional Information**

**Genotypes of flies used in article:**

**Fig. 1**

**(a**-**a’’)** *ptc*-Gal4/+

**(b**-**b’’)** *ptc*-Gal4/*UAS*-Tl^10B^

**(c**-**c’’)** *ptc*-Gal4/*UAS*-Tl^10B^; *UAS*-*GFP-IR/+*

**(d**-**d’’)** *ptc*-Gal4/*UAS*-Tl^10B^; *UAS*-*dorsal-IR^B^/+*

**(e**-**e’’)** *ptc*-Gal4/*UAS*-Tl^10B^; *UAS*-*dorsal-IR^V^/+*

**(f**-**f’’)** *ptc*-Gal4/*UAS*-Tl^10B^; *UAS*-Dronc^DN^*/+*

**(g**-**g’’)** *ptc*-Gal4/*dl^d05894^*

**(h**-**h’’)** *ptc*-Gal4/+; *UAS*-*cactus-IR-1/+*

**Fig. 2**

**(a** and **k)** *Sd*-Gal4/+

**(b** and **l)** *Sd*-Gal4/+; *UAS*-Tl^10B^/+

**(c** and **m)** *Sd*-Gal4/+; *UAS*-Tl^10B^/+; *UAS*-*GFP-IR*/+

**(d** and **n)** *Sd*-Gal4/+; *UAS*-Tl^10B^/+; *UAS*-*dorsal-IR^B^*/+

**(e** and **o)** *Sd*-Gal4/+; *UAS*-Tl^10B^/+; *UAS*-*dorsal-IR^V^*/+

**(f)** *Sd*-Gal4/+; *rpr*-LacZ/+

**(g)** *Sd*-Gal4/+; *UAS*-Tl^10B^/+; *rpr*-LacZ/+

**(h)** *Sd*-Gal4/+; *UAS*-Tl^10B^/+; *rpr*-LacZ/*UAS*-*GFP-IR*

**(i)** *Sd*-Gal4/+; *UAS*-Tl^10B^/+; *rpr*-LacZ/*UAS*-*dorsal-IR^B^*

**(j)** *Sd*-Gal4/+; *UAS*-Tl^10B^/+; *rpr*-LacZ/*UAS*-*dorsal-IR^V^*

**Fig. 3**

**(a**-**a’’)** *Sd*-Gal4/+

**(b**-**b’’)** *Sd*-Gal4/+; *UAS*-Tl^10B^/+; +/*UAS*-*GFP-IR*

**(c**-**c’’)** *Sd*-Gal4/+; *UAS*-Tl^10B^/+; +/*UAS*-Bsk^DN^

**(d**-**d’’)** *Sd*-Gal4/+; *UAS*-Tl^10B^/+; +/*UAS*-Puc

**(e**-**e’’)** *Sd*-Gal4/+; *UAS*-*cactus-IR-1/+*

**(f**-**f’’)** *Sd*-Gal4/+; *UAS*-*cactus-IR-1/UAS*-Bsk^DN^

**(g**-**g’’)** *Sd*-Gal4/+; *UAS*-*cactus-IR-2/+*

**(h**-**h’’)** *Sd*-Gal4/+; *UAS*-*cactus-IR-2/UAS*-Bsk^DN^

**Fig. 4**

**(a)** *ptc*-Gal4/+; *puc^E69^*/+

**(b)** *ptc*-Gal4/*UAS*-Tl^10B^; *puc^E69^*/+

**(c)** *ptc*-Gal4/*UAS*-Tl^10B^; *puc^E69^*/*UAS*-Bsk^DN^

**(d)** *Sd*-Gal4/+; *puc^E69^*/+

**(e)** *Sd*-Gal4/+; *UAS*-Tl^10B^/+; *puc^E69^*/+

**(f)** *Sd*-Gal4/+; *UAS*-Tl^10B^/+; *puc^E69^*/*UAS*-Bsk^DN^

**Fig. 5**

**(a** and **e)** *GMR*-Gal4/+

**(b** and **f)** *GMR*-Gal4/*UAS*-Tl^10B^; *UAS*-GFP/+

**(c** and **g)** *GMR*-Gal4/*UAS*-Tl^10B^; *UAS*-Bsk^DN^/+

**Fig. 6**

**(a)** *Sd*-Gal4/+

**(b)** *Sd*-Gal4/+; *UAS*-Tl^10B^/+

**(c)** *Sd*-Gal4/+; *UAS*-Tl^10B^/+; *UAS*-Sod1/+

**(d)** *Sd*-Gal4/+; *UAS*-Tl^10B^/+; *UAS*-Bsk^DN^/+

**(e)** *Sd*-Gal4/+; *puc^E69^*/+

**(f)** *Sd*-Gal4/+; *UAS*-Tl^10B^/+; *puc^E69^*/+

**(g)** *Sd*-Gal4/+; *UAS*-Tl^10B^/+; *puc^E69^*/*UAS*-Sod1

**(h)** *Sd*-Gal4/+; *UAS*-Tl^10B^/+; *puc^E69^*/*UAS*-Bsk^DN^

**Figure S1**

**(a)** *ptc*-Gal4/+; *UAS*-GFP/+

**(b)** *Sd*-Gal4/+; *UAS*-GFP/+

**(c)** *GMR*-Gal4/+; *UAS*-GFP/+

**Figure S2**

**(a)** From left to right: (1) *act*-Gal4/+, (2) *act*-Gal4/*UAS*-*dorsal-IR^B^*, (3) *act-*Gal4/*UAS*-*dorsal-IR^V^*

**(b)** From left to right: (1) *act*-Gal4/+, (2) *act*-Gal4/*UAS*-*cactus-IR-1*, (3) *act*-Gal4/*UAS*-*cactus-IR-2*

**Figure S3**

**(a)** *ptc*-Gal4/+

**(b)** *ptc*-Gal4/*UAS*-Tl^10B^; *UAS*-*GFP-IR/+*

**(c)** *ptc*-Gal4/*UAS*-Tl^10B^; *UAS*-Bsk^DN^*/+*

**(d)** *ptc*-Gal4/*UAS*-Tl^10B^; *UAS*-Puc*/+*

**(e)** *ptc*-Gal4/+; *UAS*-*cactus-IR-1/+*

**(f)** *ptc*-Gal4/+; *UAS*-*cactus-IR-1/UAS*-Bsk^DN^

**Figure S4**

**(a)** *Sd*-Gal4/+; *puc^E69^*/+

**(b)** *Sd*-Gal4/+; *puc^E69^*/*UAS*-*cactus-IR-1*

**(c)** *Sd*-Gal4/+; *puc^E69^*/*UAS*-*cactus-IR-2*

**Figure S5**

**(a)** From left to right: (1) *Sd*-Gal4/+, (2) *Sd*-Gal4/+; *UAS*-Tl^10B^/+, (3) *Sd*-Gal4/+; *UAS*-Tl^10B^/+; *UAS*-Bsk^DN^/+, (4) *Sd*-Gal4/+; *UAS*-Tl^10B^/+; *UAS*-Sod1/+

**Figure S6**

**(a)** From left to right: (1) *Sd*-Gal4/+, (2) *Sd*-Gal4/+, (3) *Sd*-Gal4/+; *UAS*-Tl^10B^/+

**Additional Figure**

**
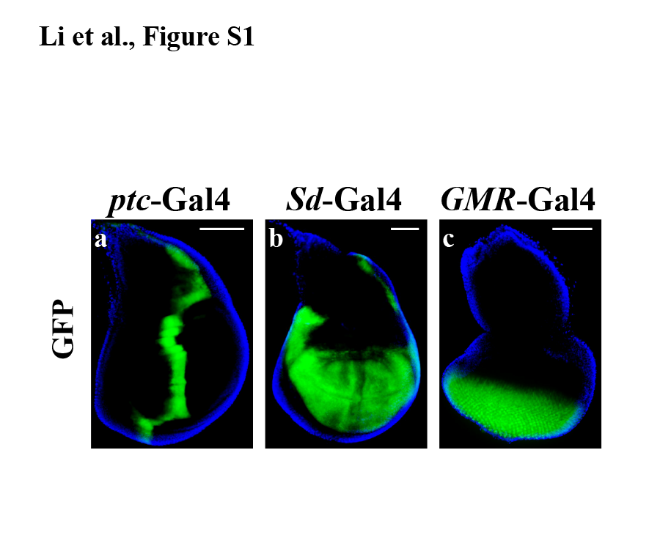
**

**Figure S1. The expression patterns of *ptc*-Gal4, *Sd*-Gal4 and *GMR*-Gal4.**

Fluorescence micrographs of third instar larval wing (**a**, **b**) and eye discs (**c**) are shown. Expression region of *ptc*-Gal4 (**a**), *Sd*-Gal4 (**b**) and *GMR*-Gal4 (**c**) are labeled by the *UAS*-GFP reporter. Scale bar: 100μm.

**
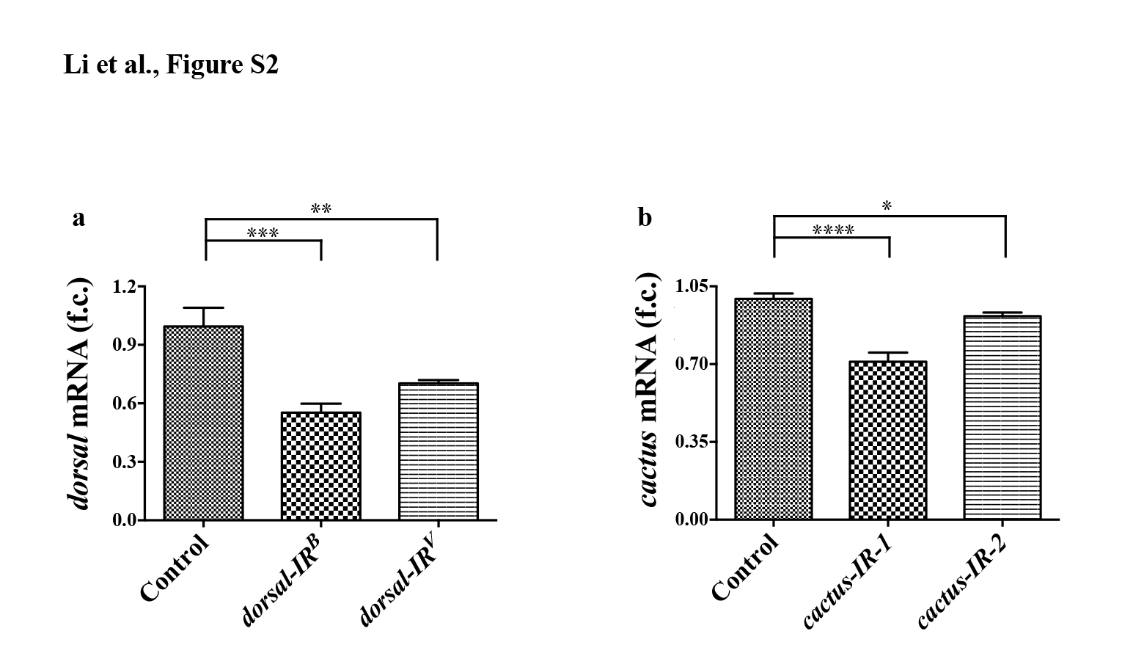
**

**Figure S2 The knock-down efficacies of *dorsal* and *cactus* RNAi lines.**

(**a** and **b**) Expression of two independent *dorsal* RNAi and *cactus* RNAi significantly decrease the level of *dorsal* mRNA and *cactus* mRNA, as measured by qRT-PCR. Total RNA of *Drosophila* third instar larval wing discs (n=10, in each group) was extracted and normalized for cDNA synthesis. Error bar indicates standard deviation. One-way ANOVA test was used to compute *P*-values, *****P*<0.0001, ****P*<0.001, ***P*<0.01, **P*<0.05.


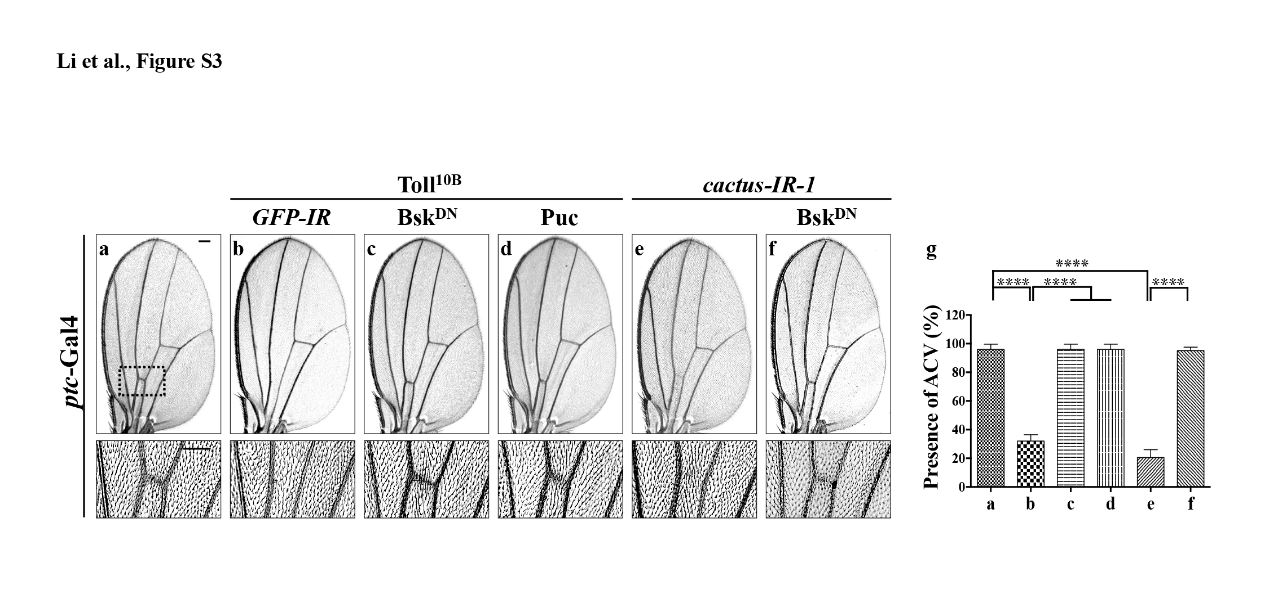


**Figure S3 JNK is required for Toll/NF-kB signaling impaired ACV development.**

Light micrographs of *Drosophila* adult wings (**a**-**f**) are shown. Compared with the *ptc*-Gal4 control (**a**), Toll^10B^-induced loss-of-ACV phenotype in adult wings (**b**), is blocked by expressing Bsk^DN^ (**c**) or Puc (**d**). Depletion of *cactus* also produces a loss-of-ACV phenotype (**e**), which is suppressed by expressing Bsk^DN^ (**f**). The lower panels show high magnification view of the boxed areas in upper panels (**a**-**f**). (**g**) Statistical analysis of ACV phenotype in adult wings (n=45 for each genotype) is shown. Error bar indicates standard deviation. One-way ANOVA test was used to compute *P*-values, *****P*<0.0001. Scale bar: 100μm.


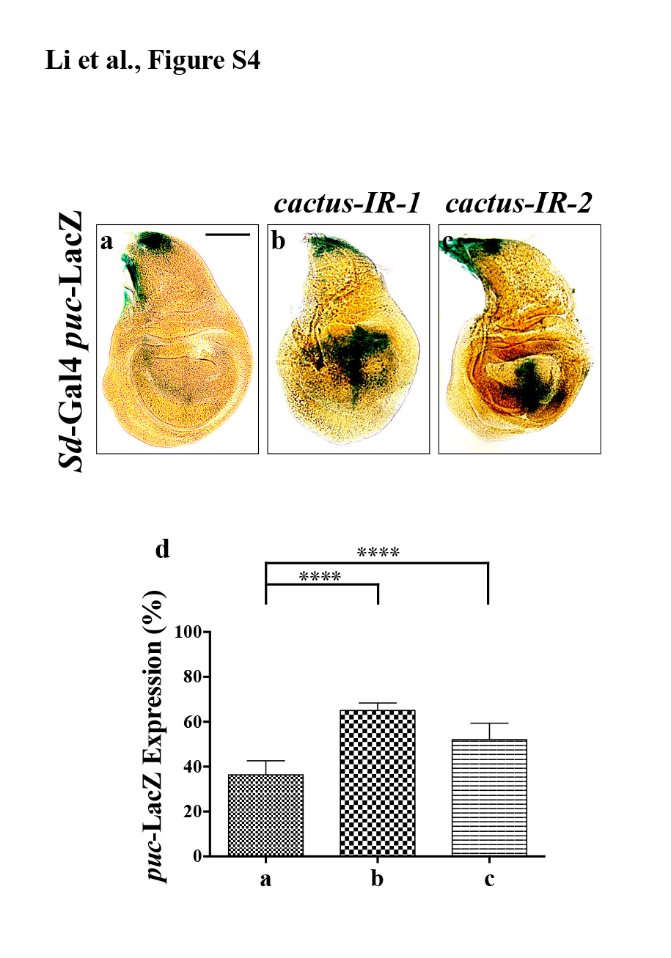


**Figure S4 JNK pathway is up-regulated by physiological activation of Toll signaling.**

Light micrographs of third instar wing discs with X-Gal staining (**a**-**c**) are shown. Compared with the *Sd*-Gal4 control (**a**), elevating endogenous Toll signaling by knockdown of *cactus* (**b** and **c**) up-regulates *puc*-LacZ expression. (**d**) Statistical analysis of X-Gal staining (n=8) is shown. One-way ANOVA was used to compute *P*-values, *****P*<0.0001. Scale bar: 100μm.


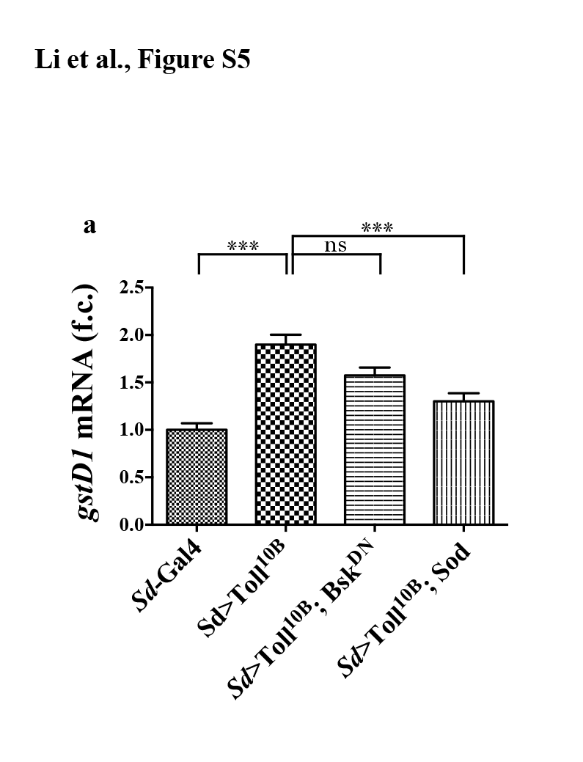


**Figure S5 Toll regulates the stress response gene *gstD1*.**

(**a**) Histogram showing the level of *gstD1* mRNA as measured by qRT-PCR. Error bar represents standard deviation from three independent experiments. One-way ANOVA was used to compute *P*-values, ****P*<0.001, ns indicates not significant.

**
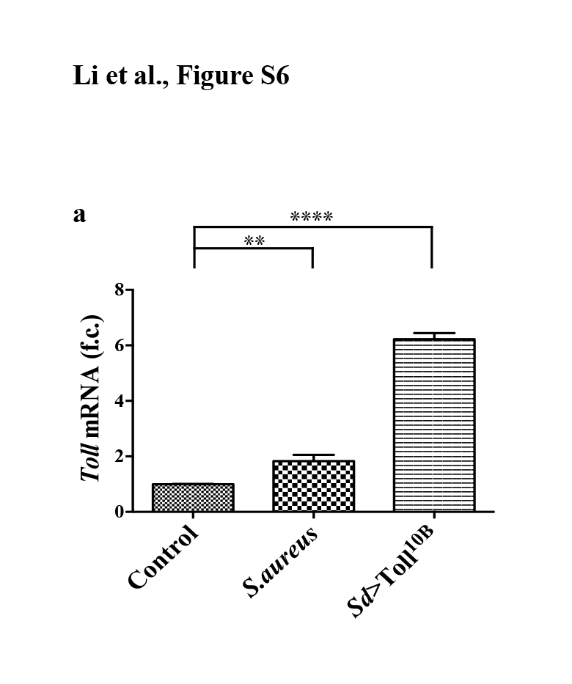
**

**Figure S6 Evaluate the level of *Toll* expression.**

(**a**) Histogram showing the level of *Toll* mRNA as measured by qRT-PCR. Error bar represents standard deviation from three independent experiments. One-way ANOVA was used to compute *P*-values, *****P*<0.0001, ***P*<0.01.
